# The magnetic cage

**DOI:** 10.1098/rsta.2023.0407

**Published:** 2024-10-09

**Authors:** E. Nasr, S. C. Wimbush, P. Noonan, P. Harris, R. Gowland, A. Petrov

**Affiliations:** ^1^ United Kingdom Atomic Energy Authority, Culham Campus, Abingdon, Oxfordshire OX14 3DB, UK

**Keywords:** STEP, magnet, HTS, REBCO, superconductor, quench

## Abstract

The Spherical Tokamak for Energy Production (STEP) requires high-field magnet designs and has therefore adopted the REBCO-based high-temperature superconductor (HTS) as its current carrier. The HTS enables the toroidal field (TF) coils to be remountable, which unlocks STEP’s vertical maintenance approach; however, remountable joints, approximately 18 GJ of stored energy and limited space down the centre of a spherical tokamak, make the TF coils the most challenging. STEP has pursued a passive approach to TF coil quench protection in order to limit coil terminal voltage. Initial results suggest that a solution may rely on tuning internal coil resistance coupled with actively powered heaters. The pre-conceptual inter-coil structure demonstrates acceptable stresses and deflections under steady-state operating conditions and preliminary fault scenarios, and loads are distributed to limit the tensile force on the TF centre rod. Finally, the HTS must operate reliably in a high radiation environment and endure high neutron fluences, ensuring commercially relevant magnet lifetimes. Initial experiments indicate that instantaneous gamma irradiation of HTS has no negative impact on current carrying capacity. Experimental programmes are underway to cold irradiate HTS to fusion-relevant fluences and to develop a method of assuring tape irradiation tolerance using oxygen ions as an analogue for neutrons.

This article is part of the theme issue ‘Delivering Fusion Energy – The Spherical Tokamak for Energy Production (STEP)’.

## 1. Introduction

To achieve a compact spherical tokamak, Spherical Tokamak for Energy Production (STEP) has adopted high-field coil designs, which make the REBCO-based high-temperature superconductor (HTS) the most suitable candidate winding material for its principal magnetic confinement systems. HTS conductors operating at around 20 K have greater thermal capacity than low-temperature superconductors (LTS) and the comparatively large margin between operating and current sharing temperature *T*_cs_ opens up the possibility of segmenting coils using resistive joints [1,2]: remountable toroidal field (TF) coils are considered by the programme to be a key enabler for STEP’s vertical maintenance approach—the TF coils remountable joints concept, and its development pathway, are discussed in an accompanying paper [3]. However, the tight aspect ratio of a spherical tokamak severely constrains the space available for these magnet systems, particularly down the centre of the reactor, and this, coupled with the need for remountable joints, makes the TF coils the most challenging.

This space constraint requires engineering current density in excess of 200 A/mm^2^ in the inboard TF winding pack, which pushes at the boundaries of HTS cable technology. Owing to the high field (approx. 17 T) in this region, the TF cables are subject to large forces that risk damaging the REBCO layer in the HTS tape, given that it is brittle and liable to delamination from its structural backing. The TF coil set also has the greatest stored energy (approx. 18 GJ) of the magnetic confinement systems, so protecting an HTS TF coil from runaway thermal transients (or ‘quenches’) is the most significant challenge [4] and has yet to be demonstrated at fusion power plant scale.

The TF coils generate substantial electromagnetic forces (100s MN), which impose challenging requirements on the tokamak inter-coil structure (ICS), especially on the slender centre rod where space for supporting structure is scarce. The centre rod also operates in the most challenging environment of the machine because space for neutron shielding is limited, resulting in very high thermal loads (10s kW/m^3^) and short inboard magnet lifetimes. This has serious implications for planned maintenance and overall availability of the plant. Replacing the centre rod vertically, which requires accurate alignment of a component 20+ m in length, and the reconnecting of cryogenic services and current leads, is a significant remote handling challenge. Realizing low resistance joints, routing current leads, winding, impregnating and assembling inboard magnets to the required manufacturing tolerances of the centre rod also require careful consideration.

This paper focuses on progress on the TF quench protection method, which is considered fundamental to the viability of the entire tokamak concept. The feasibility of the accompanying ICS is also discussed. In addition, given how crucial it is that HTS tape operates reliably in a high radiation environment and endures high neutron fluences, the progress in this area is also briefly summarized.

Finally, the poloidal field (PF) coil set is the starting point for the spatial integration of the tokamak: the positions of, and the currents within, the PF coils define the shape of the plasma. Compared with the TF coils, all the PF coils (except for the inboard divertor shaping coils) are less space limited allowing for lower engineering current densities (<100 A/mm²). The TF remountable joints also open up the possibility of positioning the PF coils inside the TF cage, allowing the PF coils to be closer to the plasma, and thus enabling the advanced divertor configurations needed by STEP. These divertor configurations require more PF coils than would be needed in an equivalent conventional tokamak, which poses a significant integration challenge. This paper also introduces the approaches and tools developed to effectively manage this integration challenge.

## 2. Toroidal field coils

### (a) TF system overview

**Figure 1.**
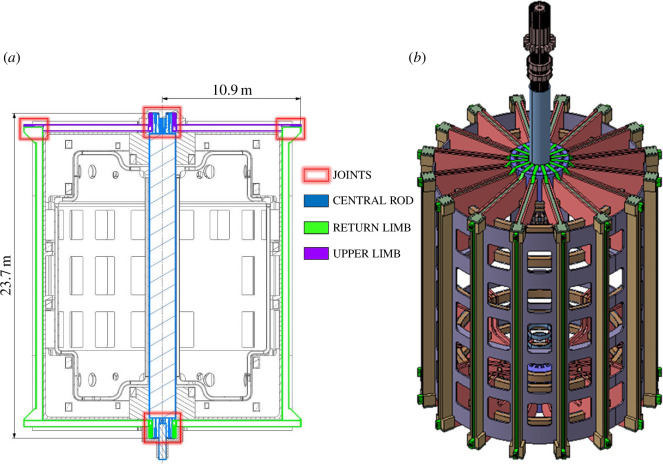
Cross section of the TF cage indicating segmentation of TF coil (*a*) and representation of the centre rod being retrieved (*b*). The CS (in light blue) can be seen wrapped around the inboard TF limbs.

The primary function of the TF coil set is to confine the plasma by providing the 3.2 T field at the plasma centre needed by the microwave heating and current drive system. The TF coil system comprises 16 ‘picture frame’ shaped coils approximately 24 m in height and 11 m in width. Each TF coil is segmented into three parts, as shown in figure 1*a*, connected by remotely operable remountable joints (see accompanying paper [3]). The remountable joints facilitate the replacement of the centre rod and enable access to in-vessel components. The centre rod comprises the inboard TF limbs, the inboard divertor shaping coils (or S coils) and the central solenoid (CS). The S coils and CS are wrapped around the inboard TF limbs. The centre rod is planned to be replaced as a single line removable unit given the limited predicted lifetimes of its magnet systems. A representation of the centre rod being retrieved is shown in figure 1*b*.

### (b) TF quench protection

Protecting large-scale HTS magnets from quench is the most significant challenge facing the technology and remains an area of active research and development [5,6]. However, this challenge is compounded in the STEP TF coil owing to three resistive remountable joints per turn, the inherent asymmetry of the coil, its tight corners and the irregular winding pack configuration necessitated by spatial constraints in the centre rod.

A quench can occur when the HTS tape locally heats up over *T*_cs_, the temperature at which the tape can no longer carry the full design current in the superconducting layer. The cable used for the TF coil system is designed to operate at 90 kA at 20 K and have a *T*_cs_ of 40 K. The 20 K difference provides a margin against AC losses, thermal gradients owing to nuclear heating, localized ohmic heating in the joints and degradation of the conductor performance due to radiation. It also allows time for action to be taken in the event of a cooling system failure. Upon heating the TF coil above *T*_cs_, some current begins to flow in the resistive components of the cable, causing it to heat up further. If heat is lost to the cooling system or adjacent coil structure more quickly than it is generated, then the coil will return to thermal equilibrium. If not, a quench, driven by the TF coils’ 18 GJ of stored magnetic energy, will occur as the temperature continues to rise until all the current flows in resistive components, forming a ‘normal zone’. If the magnet is unable to mitigate the normal zone, irreversible damage is likely to occur.

Quenches propagate slowly in HTS magnets compared with LTS owing to energy margins that are orders of magnitude higher. A quench that starts locally is unlikely to propagate quickly enough by passive means to avoid excessive local heating. It is therefore necessary to develop techniques to detect the onset of quenches and manage the stored magnetic energy to protect the TF coil set.

**Figure 2.**
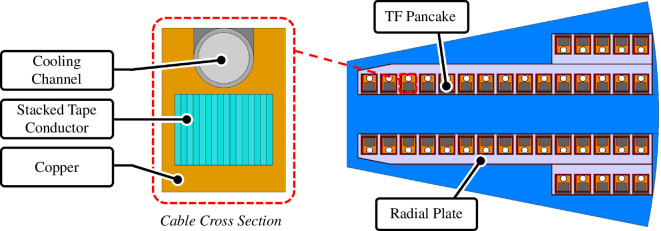
Cross section of a cable comprising a copper channel with a built-in cooling pipe and a stack of HTS tapes (left). Schematic of 40-turn TF winding pack showing four pancakes in a radial plate configuration (right).

A schematic of a proposed TF cable and winding pack configuration can be seen in figure 2. The cables in the high-field region (approx. 17 T) comprise a stack containing 230 HTS tapes with the operating current being at approximately 50% of *I*_*c*_ at 20 K. The tapes are assumed to be soldered into a copper channel with an integrated cooling pipe. A cable development programme, outlined in §5, is underway to explore various stack manufacturing methods. In addition, the manufacturing of HTS tape remains a relatively immature industry at this stage, despite significant improvements as production volumes ramp up to meet the demands of fusion [7]. To accommodate the inevitable performance drop-outs resulting from localized manufacturing defects, STEP adopts the simple approach of bundling large numbers of tapes into a single current sharing conductor, much like the stranding of LTS filaments. The resulting statistical drop-out tolerance is key to the efficient utilization of large volumes of HTS tape without the need for an unworkable degree of quality control of individual tapes. The development of non-destructive in-line methods of quality-assuring the performance of HTS tapes under the conditions required by fusion is an open challenge for tape manufacturers.

**Figure 3.**
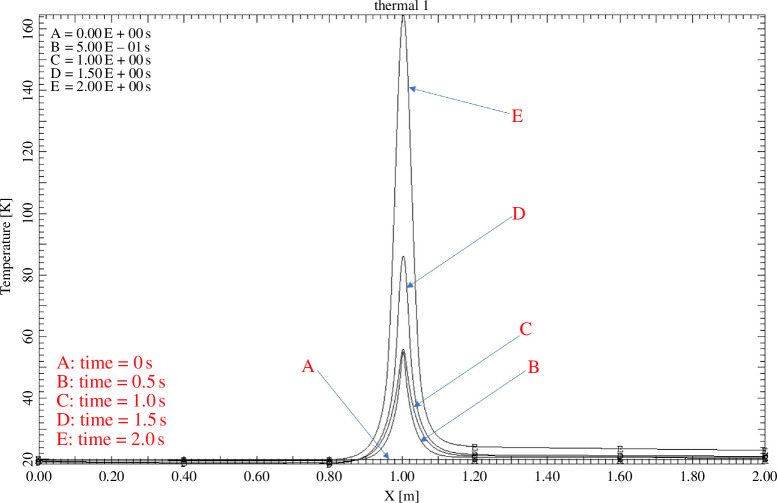
Temperature distribution along a cooled cable at various times after quench initiation.

In a one-dimensional thermal diffusion model of the TF cable, cooling is provided by 3.7 g/s of super-critical helium. A quench is initiated by a 140 J perturbation over the central 6 mm of a 2 m cable. Figure 3 illustrates the rapid temperature rise following the perturbation (applied in the first 0.5 s) and slow quench propagation. The temperature rises to approximately 160 K in 2 s if maintained at full current. To prevent the temperature from rising above 200 K, it is necessary to discharge the coil in approximately 5 s. This requires a total of 80 kV across the coil set, or 5 kV per TF coil, which is comparable with the terminal voltage of ITER TF coil pairs. However, the TF coils’ remountable joints operate in a vacuum in a highly constrained space, and in order to maintain coil terminal voltages below approximately 150 V to avoid Paschen breakdown, the STEP TF coils cannot be discharged quickly enough using traditional methods.

An alternative approach often used for small-scale HTS magnets is to omit insulation between turns altogether. During energization, current can flow radially through the magnet structure between the turns. Eventually, in a non-segmented coil, the radial resistive voltages produced will force all the current through the superconducting circuit. If part of one turn becomes resistive the resulting electric field drives current radially through the magnet structure and through turns that are still superconducting. This reduces local cable heating and helps spread the normal zone quickly by distributed heating of the coil structure. The potential for self-protection has been demonstrated many times in small magnets [8,9].

However, STEP’s remountable joints add resistance exceeding 100 nΩ in series with the superconducting cable in a TF coil. This is higher than the radial resistance of the TF coil stainless steel structure and additional resistance, or ‘partial’ insulation, must be added between the cables in the winding pack to ensure most of the current flows azimuthally. The resistivity of this partial insulation layer, which in the analysis presented below includes the contact resistance between the insulating material and interfacing structures, can be tuned such that current transfer between adjacent turns can still occur, the radial resistance is sufficiently high to allow coil energization despite the resistance of the remountable joints, and the voltage across the coil terminals is kept below that required for Paschen breakdown. The integrity of the bond between the partial insulation layer and interfacing structures is crucial for uniform energy deposition and will be subject to extensive experimental validation and process control development.

**Figure 4.**
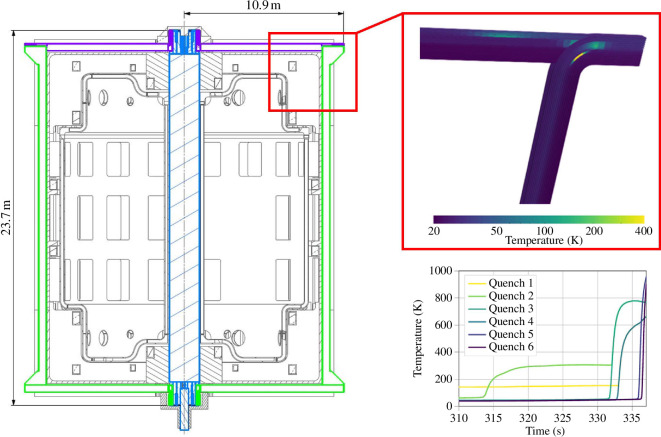
Raccoon model of a toroidal field coil. Hotspot evolution is shown. The local temperature rises to over 800 K in approximately 340 s, at which point the simulation is terminated.

Raccoon is an electrical network solver based on experience with JackPot [10,11], optimized by Little Beast Engineering and has been used to investigate hotspot development. An example is seen in figure 4. The TF coil modelled considers the cable concept described above and the 40-turn winding pack configuration illustrated in figure 2. The preliminary fault scenarios assumed are total loss of power and coolant supply leading to an open-circuit discharge.

In this example, the radial resistivity of the coil is such that the discharge time constant is approximately 26000 s, as calculated by Raccoon. If current sharing occurs somewhere in the coil, but the radial currents do not result in the coil recovering full superconductivity, such a long time constant means it cannot be de-energized quickly enough to avoid damage. Radial current will tend to flow where current sharing is greatest and the azimuthal resistance is highest. This occurs at flux density hotspots, which in a single largely planar coil, as modelled here, are at the corners, as can be seen in figure 4. To date, no satisfactory solution has been found that works within the required resistivity constraints and the problem is exacerbated when the whole 16-coil set is considered.

**Figure 5.**
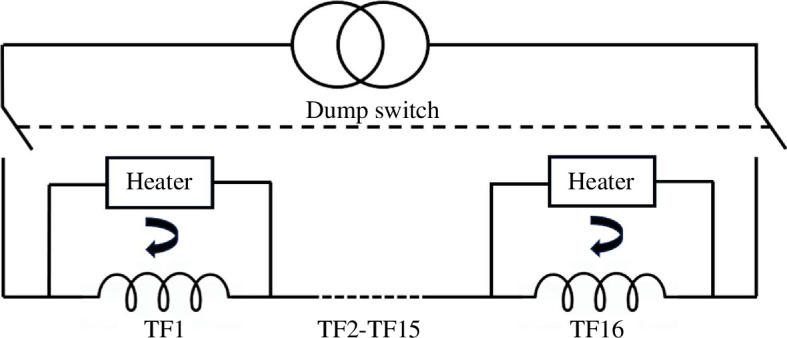
Dump switches are used to transfer stored magnet energy to heaters embedded in the coil structure.

Furthermore, in a completely passive system there is no guarantee that all the TF coils will de-energize at the same rate and in the same way, which could lead to significant out-of-balance toroidal forces. The STEP TF coil may require some action to be taken to safely dissipate the stored energy without creating hotspots. The STEP programme is currently investigating the use of the TF magnet’s stored energy to power heaters embedded within the coil structure. A simplified schematic can be seen in figure 5.

The heaters could be connected to the coil terminals, so when a dump switch is opened current circulates around each coil/heater pair, warming up the coils uniformly to *T*_cs_. The aim is to tune a combination of internal coil and heater resistances to deliver TF system de-energization without excessive hotspots and mechanical load imbalance, while also keeping the peak voltage below the Paschen minimum. This approach requires a reliable method of detecting the onset of thermal runaway.

### (c) TF quench detection

Various quench detection methods have been proposed for HTS magnets, for example:

— Voltage taps.
— Fibre optics (discrete fibre Bragg gratings and continuous based on Rayleigh back-scattering).
— HTS circuits that transition from superconducting to normal earlier than the coil cable.
— Ultrasonic waveguides.
— Radio frequency (RF) coolant density measurement.
— Coolant mass flow rate, pressure and temperature.
— Inductive quench antennas.
— Acoustic thermometry.
— Diffuse-field ultrasonic methods.
— Hall sensor arrays.

The traditional voltage tap method is unlikely to work in a large HTS coil system: slow normal zone propagation and rapid heating mean damage will be caused before a measurable voltage develops in the electrically noisy reactor environment. Several of the proposed methods require the installation of additional hardware in places where space is at a premium. It is also required that sensors embedded within the coil pack can tolerate high mechanical stress without damage whilst also surviving radiation damage for the life of the coil. The STEP TF coil system will contain around 45 km of cable and quench hotspots that have length scales of the order of 10 mm (figure 3). Methods with discrete, localized sensors are likely to require too many data channels to be useful. Acoustic thermometry [12,13] and diffuse-field ultrasonic methods [14] are likely to be difficult to interpret given the size and complex geometry of the STEP TF magnets. While it is too early to rule out any technique, non-invasive and distributed measurement methods are preferred.

For example, RF coolant density measurement techniques to detect quenches in LTS cables were developed for ITER in the 1990s [15]. These involve helium density measurements using resonant frequency sensors (660 MHz) and super-high frequency (37 GHz) interferometry. Helium density measurement sensitivity was in the range of 0.5–2.5 g/m^3^. However, ITER did not adopt the technique owing to attenuation losses caused by the nature of the ITER cable cooling channels, whereas STEP cables could adopt a smooth wall cooling channel. Furthermore, the minimum quench energy for HTS coils is higher, so less sensitivity is required. While the use of RF methods in superconducting magnet diagnostics has been minimal, recent experiments with HTS have shown its potential as a robust and inexpensive option for the detection and localization of quench hotspots [16].

Results from simulations to date indicate that using an RF method could allow detection of heating in the HTS stack well before *T*_cs_ is reached, even when operating at a low *I*_*c*_ margin. Having a large detection margin would allow sufficient time to initiate quench protection measures. A proof of principle test programme is underway, starting with a pipe and heater system at room temperature and building up to a full-size cable operating at 20 K in the background field at the SULTAN test facility, to further investigate this approach. Investigations are also underway to integrate this technique with the cryogenic cooling manifolds and remountable joints.

## 3. Poloidal field coils

### (a) PF system overview

**Figure 6.**
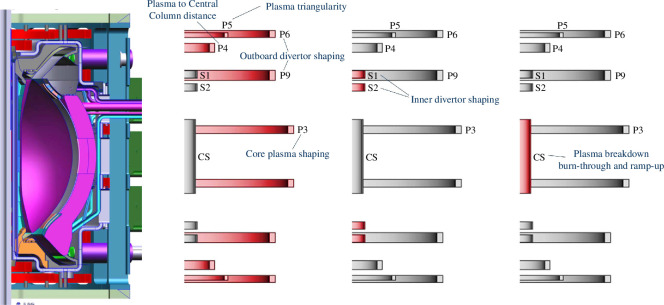
PF coil set and summary of plasma control functions of the various coils.

The PF coil set consists of seven (P3, P4, P5, P6, P9, S1 and S2) pairs of coils (mirrored around the midplane of the tokamak) and a CS, as shown in figure 6. All PF coils are assumed to be HTS except for the CS, which has adopted a resistive design. The resistive CS can tolerate higher neutron fluxes than an HTS coil and so it improves the overall lifetime of the centre rod. The primary roles of the PF coils are as follows:

— The CS delivers approximately 9 Vs of flux using a unipolar current swing (because resistive heating prevents the coil from carrying significant steady-state current), which is predicted to be sufficient to achieve plasma breakdown, burn-through and ramp-up of the plasma current inductively to approximately 1–2 MA. The required magnetic field null for plasma breakdown is achieved using a combination of currents in the outer P3–P9 coils. The solenoid is energized just before the start of the plasma scenario and will be completely de-energized at the end of the inductive current drive phase, between 3 and 10 s later; at this point, the CS is assumed to no longer provide inductive current drive during start-up operations with the diverted, full-bore plasma now a suitable target for the non-inductive current ramp-up phase that will use a high-power microwave system [17,18].
— During the remainder of the plasma scenario (non-inductive current ramp, plasma flat-top and current ramp-down) the P3–P9 coil currents are used to achieve the required plasma shape, including divertor formation. Generally, there is sufficient space available for these coils to allow the design current density to be in the typical range for HTS coils.
— The S1 and S2 coils are used to tailor the shape of the inner legs of the divertors. The limited space available for these coils means that the required current density is high, and the space afforded for neutronic heating is low, which is challenging.
— For some of the coils, including the CS, there is also a secondary role of assisting with plasma control through low amplitude field modulation.
— Passive vertical stabilization of the plasma current and active control of plasma instabilities is achieved using separate dedicated coil sets (control coils) not described in this paper.

The location of the coils, their radii and the total current carried in them is fundamental to the definition of the plasma design basis. Therefore, the PF coil set configuration is the starting point for the integrated design of the tokamak. Some of the methods and tools for achieving rapid iteration of the STEP concept design are presented below.

### (b) Data-centric design process

Three principles form the basis of the PF coil set design process to ensure rapid iteration cycles:

— Centralized design data—well-defined, consistent and version controlled to enable efficient analysis and collaboration. For example, a schema has been defined for a machine and human-readable datafile capturing key coil set parameters, which can interface directly with analysis and CAD tools.
— Knowledge capture and reuse—capturing learning through a combination of simple models, design rules and data objects enabling learning to be exploited at earlier stages of the process and minimizing the repetition of the evaluation. An example is the capture of spatial constraints from the plasma to each coil to allow spatial integration independent of variation of plasma shape.
— Integrated design tools—facilitated by centralized design data with the goal of utilizing a high degree of automated tools, maximizing the value added by engineers by eliminating time-consuming pre-processing and data handling tasks. The Analytical Python Electromagnetic Coil Simulator (APECS) code presented below is the most important of these tools at this stage of STEP design maturity.

### (c) APECS toolbox development

In the early stages of concept iteration, multiple plasma scenarios and magnet requirements sets were explored. This had to be done at pace with the development of APECS, which uses Biot–Savart equations to calculate the magnetic field of the magnets on STEP. It deals efficiently with magnetostatic calculations in PF (circular, axisymmetric) and TF (picture-frame with straight limbs) magnet systems. The tool also calculates mutual inductances and the resulting voltage response of individual magnets.

Most notably, APECS provides quick estimates of the minimum physical size of each magnet system, assuming a range of superconducting cable types. At the time of writing, the greatest limitation of the algorithm is that it only considers the magnetic fields and does not consider stress and strain or perform any optimization on the magnet cable. This has been published and discussed in greater depth in [19].

## 4. Inter-coil structure

The key functions of the ICS are to maintain the positions of the magnetic coils, to withstand operating loads and to ensure deflections do not cause clashes with interfacing systems. While initial design iterations focused on a more traditional ‘TF-led’ structure, a ‘PF-led’ approach has been adopted to enable STEP’s vertical maintenance strategy: the ICS surrounds the PF coils with all other supports mounted to this PF superstructure.

**Figure 7.**
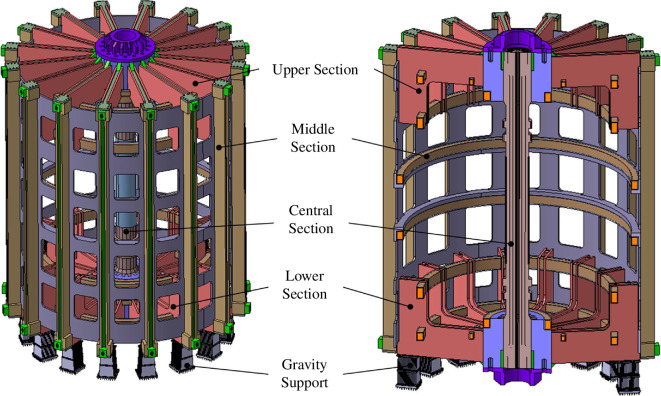
Overview of the inter-coil structure, highlighting the five distinct modules.

The ICS is split into five distinct modules, as shown in figure 7. The structure is assumed to be constructed from 316 LN stainless steel and has been designed to use commercially available plate thicknesses. For maintainability, each module of the structure can be demounted and removed vertically, with sub-assembly masses being limited to 1500 t.

Preliminary global static structural analysis of the ICS set out to demonstrate acceptable stresses and deflections limited to steady-state flat-top operating conditions and, as such, it does not include the CS coil. The analysis also served to iterate the design of the ICS with the objective of transferring the bursting loads as much as possible from the horizontal TF limbs through the outer TF legs to limit the tensile loads on the TF centre rod. The flat-top model uses cyclic symmetry to assess a one-sixteenth segment of the ICS with a coarse mesh and simple, bonded contacts. While the TF pancakes were represented as rectangular cross sections bonded into the stainless steel casings, the fidelity of the model was such that cable material compositions were simply homogenized. The loads were applied in timesteps; the first load condition applied was thermal, reducing the temperature of the structure to 20 K. The second timestep applied the electromagnetic loads, which were applied as a body force density to the coil structure.

**Figure 8.**
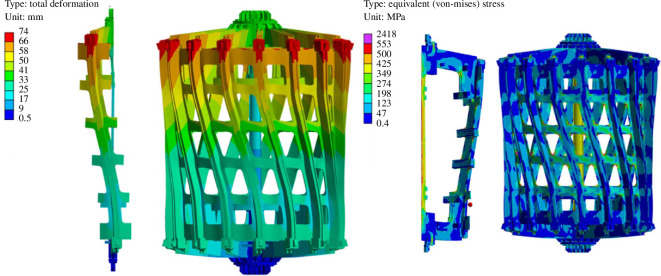
Total deformation plot and von Mises stress plot under thermal and flat-top electromagnetic loads.

An overview of the total displacement and global von Mises stress has been included in figure 8. Given that the ICS is supported by gravity supports, the maximum total displacement (a combination of vertical, radial and toroidal deflections) of 74 mm is shown at the crown of the structure on the left of figure 8. Total radial and vertical deformation is principally caused by thermal contraction and is similar in magnitude to that of ITER. However, toroidal displacement is approximately twice that of ITER.

**Figure 9.**
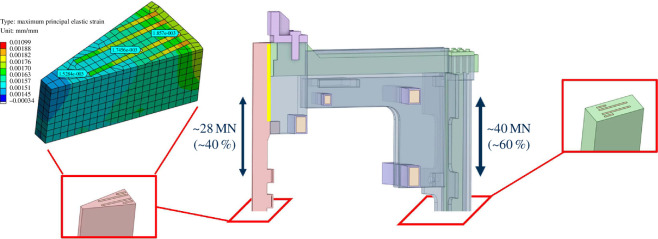
Force distribution through the inter-coil structure showing top end of a TF coil (middle). Simple representations of winding pack cross sections shown on inboard (wedge-shaped) and outboard TF limbs. Mid-plane strain analysis of inboard winding pack (containing four radial pancakes) showing maximum principal strain on HTS cables at <0.2% (left).

Critical components of the structure are below ITER’s 550 MPa limit (note, the maximum von Mises stress occurs at discontinuities in the outboard side of the structure (the point of maximum stress is indicated by the small red ball on the right of figure 8) - these are not of significant interest in the global model and would require further geometry optimization and mesh refinement to study further). The analysis has also shown the centre rod carries a significant amount of stress. Large loads are transferred through a comparatively slender centre rod and the design of the ICS has been iterated to distribute as much load as possible outboard, as shown in figure 9.

The inboard TF coil winding pack, shown on the left-hand side of figure 9, is subjected to a challenging structural load case during flat-top operation. Early analysis shows that in the midplane of the centre rod the axial strain (<0.2%) and shear stress (<5 MPa) experienced by the HTS tape are within assumed limits [20]. Work is underway to quantify screening currents [21] induced during transient events leading to non-uniform current distribution and stress concentrations in the HTS tape, which could be a leading cause of damage.

Approximations of the expected bounding fault scenarios were also assessed using a full 16-segment model. The most extreme case assessed was a conservative representation of a TF coil quench with a single TF completely de-energized while all others remain at full power, resulting in large asymmetric loads. The quench impulse loading has been applied to the model as a static structural load. To limit computational cost, the structure was modelled using bonded contacts throughout.

**Figure 10.**
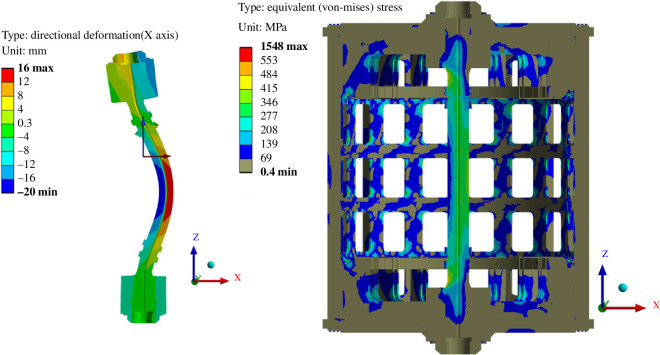
Fault load overview: exaggerated radial deflection of centre rod (left); stress plot of whole structure (cross section through the centre of the machine) (right).

The stress results shown in figure 10 indicate the structure would not fail elastically under the applied asymmetric fault load; critical components of the structure are below ITER’s 550 MPa limit. The deflection of the centre rod, however, shown to the left of figure 10, is significant and emphasizes the challenge of maintaining clearances with interfacing components tightly packed in the central column of the machine. Note that the centre rod mainly comprises 16 inboard TF limbs and the model optimistically assumes these are all perfectly bonded together: representative connections will affect the stiffness of the centre rod structure and therefore the response. However, the CS coil is assumed to be wrapped around the inboard TF limbs, and therefore insulation between TF wedges is not envisioned, which is likely to simplify the design.

## 5. Technology development programme

The successful realization of the unprecedented scale HTS magnet designs outlined in the previous sections hinges on a robust model validation approach underpinned by an extensive technology development programme, particularly around quench protection. Early stages of this programme have targeted critical technological open questions around remountable joints development (see [3]) and radiation impact on HTS materials, while targeted test programmes are under development addressing specific critical aspects of the concept design.

### (a) Magnet materials test programme

Three high-level needs for magnet materials development were identified early in the programme and work was initiated to address them:

— Quantification of radiation tolerance of HTS REBCO tape.
— Selection/development of radiation-tolerant insulation material.
— Development of targeted resistivity partial insulation material.

The radiation tolerance of available HTS tapes is being considered as a fixed, but so far unknown, quantity around which the machine and its maintenance strategy will need to be designed. In this regard, the programme is concerned with the effects of the instantaneous flux of irradiation and the lifetime accumulated fluence the HTS tapes can withstand.

STEP has begun a programme [22] to test the irradiation resilience of the HTS tapes under consideration for its magnets. Initial experiments have, so far, indicated that instantaneous gamma irradiation of HTS tapes has no negative impact on current carrying capacity at 77 K—an encouraging result [23]. Ion irradiation experiments, by contrast, initially showed an increasing and instantaneous current carrying capacity degradation with increasing flux [24]. Subsequent investigations, however, have suggested that this may be owing to sample heating through ion bombardment rather than an intrinsic ion-superfluid interaction [25].

Given the limited availability of fusion-relevant irradiation data [26], STEP’s experiments have been designed to inform STEP’s choice of HTS tape for its magnets and determine a cost-effective method of assuring tape irradiation tolerance during magnet manufacture. A filtered oxygen ion irradiation experiment [27] seeks to mimic the damage profile of a fusion neutron spectrum using 20 MeV oxygen ions, chosen as SPECKTRA-PKA [28] simulations show that 60–70% of lattice damage from neutron irradiation in REBCO is owing to oxygen knock-ons [29] (i.e. oxygen ions displaced by neutrons that then go on to impact other ions in the lattice). The oxygen ions are to be passed through a Steinbach-style energy filter [30] to defocus the irradiation’s Bragg peak and increase the oxygen ion damage-to-implantation ratio, limiting the effect of oxygen ion implantation on REBCO’s superconducting properties. The programme also includes STEP’s high-neutron fluence cryogenic irradiation of superconductors (Hi-CrIS) experiment with Centrum Výzkumu Řež (CVŘ). Hi-CrIS will irradiate HTS tape samples to fusion-relevant fluences using cadmium-shielded fission spectrum neutrons, while maintaining them at STEP’s operating temperature of 20 K. This will provide validation of the oxygen ion irradiation experiment.

The assumed ground insulation material is a radiation-resistant cyanate ester/epoxy blend, as used on ITER [31]. By adjusting the proportion of cyanate ester, it is envisaged that the radiation tolerance of the insulation can be raised such that the REBCO tape remains the lifetime-limiting factor. Only if this proves not to be the case will a development programme be undertaken to find a new insulator material. By contrast, the cable inter-turn insulation material of the TF coils is a subject of development in order to achieve a resistivity value that successfully balances the protection of the coil in the event of a quench against charging capability. This is found to be beyond what is achievable using metallic insulation necessitating the adoption of some form of novel material.

The development of insulation bonding methods and process control will also be part of the materials development programme. For example, the failure of bond planes between the partial insulation layer and the cable or its supporting structure would cause potentially damaging non-uniform heating in the event of a rapid coil discharge. The magnet design will aim for compressive and minimal shear stresses in bond planes and, given the combination of structural and thermal stresses with complex coil geometries, the strategic placement of partially insulating materials.

### (b) Small coil, short cable and model coil test programmes

The proposed technology development pipeline runs through three distinct areas of focus. Almost in parallel, a small coil test programme will provide essential practical know-how and skill-building in the area of HTS magnet design and manufacture, enabling fast-paced low-cost optioneering and real-world development of novel coil components such as partial insulation and quench heaters, while a short cable test programme will focus on the manufacture, test and optimization of full-scale short-length cables for each coil type, verifying their performance to the required specification under the expected operating conditions. For example, non-uniform current distribution can result in stress and strain concentrations in the HTS tape, particularly when induced by screening currents generated by changing magnetic fields. The large forces experienced by the TF winding pack are being actively studied in cables of various construction, including vacuum pressure impregnation, pre-tinning and ‘wet winding’ techniques. These full-scale cables will also enable the development and testing of the novel quench detection methodologies previously outlined. Together, these two test programmes then feed into a sub-scale model coil test programme that will provide validation of the concept for each of the coil sets at a representative scale.

## 6. Closing remarks

Preliminary work on STEP’s principal magnetic confinement systems has identified the main challenge areas which focus around the TF coil system. The TF coils are planned to be remountable and are likely to be the largest HTS magnets that have ever been built; protecting these large coils from quenches is the most significant technical challenge. Other challenges include the high neutron fluences these magnets need to endure to demonstrate commercially relevant operational availability. A magnet technology development programme has been set in motion to start tackling these critical challenges. Collaboration will be essential in developing these technologies, and the authors are actively seeking opportunities to work with industry and the wider magnets community.

## Data Availability

Data can be accessed via the UKAEA Open Data Register (to be set-up following initial submission). To obtain further information on the data and models underlying this paper please contact PublicationsManager@ukaea.uk.
